# The Investigation of Tramadol Dependence with No History of Substance Abuse: A Cross-Sectional Survey of Spontaneously Reported Cases in Guangzhou City, China

**DOI:** 10.1155/2013/283425

**Published:** 2013-09-12

**Authors:** Haoran Zhang, Zhimin Liu

**Affiliations:** ^1^School of Public Health, Peking University, Beijing 100191, China; ^2^National Institute on Drug Dependence, Peking University, 38 Xueyuan Road Haidian District, Beijing 100191, China

## Abstract

The study was to survey and assess the drug dependence and abuse potential of tramadol with no history of substance abuse. Subjects of tramadol dependence with no prior history of substance abuse were surveyed by interview. Physical dependence of tramadol was assessed using 10 items opiate withdrawal scale (OWS), and psychological dependence was assessed by Addiction Research Center Inventory—Chinese Version (ARCI-CV). Twenty-three male subjects (the median age was 23.4 ± 4.1 years) referred to the addiction unit in Medical Hospital of Guangzhou with tramadol abuse problems were included in this cross-sectional study. The control group included 87 heroin addicts, 60 methamphetamine (MA) abusers, and 50 healthy men. The scores of OWS of tramadol were 0.83–2.30; the mean scores of identifying euphoric effects–MBG, sedative effects–PCAG, and psychotomimetic effects–LSD of ARCI were 8.96 ± 3.08, 6.52 ± 3.25, and 6.65 ± 2.50, respectively, *F* = 4.927, *P* < 0.001. Scores of MBG scale in tramadol did not differ from those in heroin and MA groups (*P* > 0.05) but were higher than those in healthy men (*P* < 0.05). Tramadol with no history of substance abuse has a clear risk of producing high abuse potential under the long-term infrequent abuse and the high doses.

## 1. Introduction

Tramadol, marketed in Germany by Grunenthal since 1977, is a centrally acting analgesic with weak *μ*-opioid agonist properties and inhibition of norepinephrine (NA) and serotonin reuptake. The drug is most widely used in moderate to severe acute and chronic pain. It has been postulated that tramadol achieves its analgesic activity from an M1 metabolite with potent opioid properties and through inhibition of reuptake of monoamines [1]. Prior to its United States (US) approval in 1995, tramadol was marketed in Europe for approximately 20 years with little evidence of abuse. The abuse probability of tramadol in USA was 2-3/100,000 and then declined to 1/100,000 [2]. World Health Organization (WHO) attached great importance to the abuse and dependence of tramadol, and four estimates were done. From 1992 to 2006, tramadol dependence was evaluated for four times by Expert Committee on Drug Dependence (ECDD) [3–6]. Because the available information was still not enough, it could not decide to control tramadol internationally. Although tramadol abuse is a low-level worldwide, the nonmedical purpose abuse of tramadol was popular in China since its first marketing in China in the early 1990s as a noncontrolled analgesic, especially in opiate addicts and adolescents. In order to prevent the abuse of tramadol and manage the clinical application of tramadol, in 2007, the State Food and Drug Administration of China issued the newest version of measures for psychotropic drugs administration and tramadol was controlled as the second category of psychotropic drugs [7]. After that, the incidence of tramadol abuse has declined to a low level. According to the report of National Drug Abuse Monitoring, the proportion of tramadol use among drug abusers increased from 0.2% in 2004 to 16.0% in 2006; the trend of tramadol use varied very smoothly from 2007 to 2009; however, the proportion of tramadol use among drug abusers declined sharply from 13.3% in 2009 to 3.4% in 2011 [8–11]. However, it is worth noting that tramadol abuse was still prevalent in some regions, and tramadol abuse shows a property of regional distribution. Among those, the percentage of tramadol use was highest in South China (5.1%), and tramadol popular use in Guangdong province was still relatively high [11]. According to statistics of Drug Monitoring Center of Guangdong Province, the number of tramadol abusers increases from 11 persons in 2004 to 4,492 persons in 2006 in Guangdong province [12]. Based on these reasons, all tramadol patients were recruited in Guangzhou city, Guangdong Province.

Tramadol is generally considered to be safe and thought to have minimal potential for abuse. Despite a range of studies, still no consensus data exist on the dependence potential of tramadol. Studies from animal experiment hold controversial finding, one is that tramadol was not likely to induce tolerance and physical dependence in mice [13] and the other is that conditioned place preference (CPP) in rats was produced [14, 15]. Though tolerance and dependence were not described after repeated administration of tramadol in human [16, 17], case reports that tramadol can lead to dependence continue to emerge [18, 19]. It was suggested that the abuse liability of tramadol may be greater than hitherto assumed [20].

Hence, data for the dependence and abuse potential of tramadol are conflicting, and important information is still missing regarding cases of tramadol dependence and abuse in China. The objective of this study was to evaluate drug dependence and abuse potential of tramadol, using tramadol abusers spontaneously referred to the addiction unit of Medical Hospital in Guangzhou city between July 2012 and December 2012.

## 2. Methods

### 2.1. Ethical Statement

The study was approved by the ethical committee of Peking University Health Center (Grant number IRB00001052-10026). Before the interviews were conducted, subjects were informed about the purpose of this study and confidentiality of all information they would provide. Respondents' consent was gathered. The survey was conducted in an isolated room, and the survey procedures were designed to protect privacy of subjects by allowing for anonymous and voluntary participation. Participants in the study could stop at any time if they desired.

### 2.2. Subjects

Identification and collection of relevant cases were conducted from 1 July 2012 to 1 January 2013. All available information in the included tramadol cases was registered: age, sex, occupation, marital status, educational status, concomitant medication, prescribed and ingested doses of tramadol, duration of tramadol abuse, and other important information. Subjects reported to have a history of continuous tramadol abuse for 7 days in a week or those who had life-time used tramadol for 6 months at least were further reviewed by the American Psychiatric Association Diagnostic and Statistical Manual of Mental Disorders, fourth revision (DSM-IV). Only subjects who fulfilled these criteria were finally included in the final analysis. Though the identification of tramadol dependence in this study was conducted based on DSM-IV criteria, the diagnosis in the detoxification unit has no such information. Generally, patients in the drug dependence agencies may require tramadol detoxification or dose reduction.

The inclusion criteria were (1) male; (2) 18 years old or above; (3) the primary abused drug of choice was tramadol; (4) willing to sign an informed consent; and (5) adhered sufficiently to the study protocol. Meanwhile, the exclusionary criteria were made: (1) unwilling to participate or could not provide written informed consent; (2) could not discriminate the primary drug of abuse; (3) severe psychotic disorders; (4) severe body diseases (e.g., prostrate disease of liver or kidney); (5) be apt to drop out; and (6) deficiency in or lack of language and comprehensive problems. The criteria for controls were (1) men aged 18 and above; (2) for healthy controls, without a history of drug abuse before the study and no evidence of drug abuse during study; (3) for heroin addicts and methamphetamine abusers, predominate drugs of choice were heroin and methamphetamine in the baseline survey, respectively; (4) provided informed consent. Overall, a total of 23 eligible tramadol addicts, 85 heroin addicts, 60 methamphetamine addicts, 45 smokers, and 50 healthy controls were recruited.

### 2.3. Data Collection and Measures

The study was a cross-sectional survey. Before the formal survey, a pilot trial was administrated. Researchers participated in the investigation were trained by empirical experts on specific aspects of survey techniques. Each sample was briefly informed about the purposes of the investigation, in order to seek their support and cooperation. After that, a written informed consent was obtained from all participants. A questionnaire with detailed baseline variables was offered by National Institute on Drug Dependence, Peking University. Current sociodemographic characteristics, history of substance use, and information of ARCI were obtained from a face-to-face interview. All finished questionnaires were reviewed by researchers for completeness and consistency. 

Physical dependence of tramadol was assessed using 10 items opiate withdrawal scale (OWS), which composed of 10 symptoms/signs. A 4-point scale was used to rate each symptoms/signs: zero (0), mild (1), moderate (2), and severe (3). Subjects were asked to rate their symptoms according to severity of previous experienced tramadol withdrawal. Psychological dependence was assessed by association test of Addiction Research Center Inventory–Chinese Version (ARCI-CV) [21]. The 40-item ARCI is a true-false questionnaire with three empirically derived scales that are sensitive to the effects of a variety of classes of abused drugs. The derived scales were the MBG (morphine-benzedrine group), a measure of drug-induced euphoria; the PCAG (pentobarbital-chlorpromazine group) scale, which measured sedation; and the LSD (lysergic diethylamide) scale, which measures dysphoria and somatic symptoms. The same method which priority evaluated drug dependence of dihydroetorphine and buprenorphine was used in this study. 

### 2.4. Statistical Analysis

Statistical analysis was completed using the SPSS computer software for Microsoft Windows (SPSS Inc, Chicago, IL, USA). Quantitative symmetrically distributed variables were described by using the mean and standard deviation, whereas quantitative variables with a skewed distribution were described by using the median. Nonparametric statistical tests were undertaken when the distribution was not normal. Descriptive statistics were used to examine distributions of demographic and history of drug abuse. Differences among subgroups of ARCI were explored using *t*-test or one-way analysis of variance (ANOVA). All tests of statistical significance were two-tailed and used an alpha level of 0.05.

## 3. Results

### 3.1. Characteristics of Demographic and History of Drug Use

Demographic characteristics and history of drug use were summarized in Tables 1 and 2. At baseline, no significant difference was observed in respondent's average age, educational status, marital status, ethics, and occupation (*P* > 0.05) across groups. 

Of the 23 tramadol patients, the median age was (23.4 ± 4.1) years and ranged between 18 and 34 years and 73.9% (17/23) of cases were lower than 25 years. 87.0% (20/23) of the sample had no previous history of drug abuse before tramadol abuse, and 90.0% (21/23) were on tramadol alone or 10.0% on poly drugs. The drugs most frequently used in combination with tramadol were cough syrup (5 subjects) and methamphetamine (MA) (3 subjects). The median dose was 750 mg/per time at frequency and 2000 mg/per time at most. The median duration of tramadol use was 61.8 ± 30.2 months (ranged from 5 months up to 112 months). The median age of first tramadol use was 18.3 ± 4.0 years. All 23 tramadol users reported that the route of administration was oral method. Instigating by friends or other acquaintances was the most reason of tramadol use for 23 cases, which accounts for 60.9% (14/23), and there were 39.1% reported that the reason of tramadol use was curiosity or simulating. As for the source of tramadol, the top three were private clinic or pharmacy (12/23), normal pharmacy (6/23), and black market (6/23). 69.6% (16/23) of subjects have at least one time or above admission to detoxification or drug reduction.

### 3.2. Measure of Physical Dependence

As mentioned in the methods, physical dependence was rated by scores of 10 items opiate withdrawal scale (OWS). In total, the range of mean scores of OWS scale was 0.83–2.30. Of those, 95.7% (22/23) of the patients had at least one seizure. Ratings on the insomnia (2.30 ± 0.93), yawning (1.83 ± 0.98), and runny eyes (1.61 ± 1.23) were the top three increased by tramadol. However, the differences of ratings of each sign/symptom between tramadol and heroin did not reach statistical significance (*P* > 0.05). 

### 3.3. Measure of Psychological Dependence

Table 3 details the measure of psychological dependence of tramadol and comparators. The mean scores of three scales in identifying euphoric effects—MBG, sedative effects—PCAG, and psychotomimetic effects—LSD of ARCI were 8.96 ± 3.08, 6.52 ± 3.25, and 6.65 ± 2.50, respectively. One-way analysis of variance indicated that the variation in 3 scales was significant (*F* = 4.927, *P* < 0.001). On the all three scales of ARCI measures, tramadol produced a significantly greater effect than healthy placebo (*P* < 0.05). On the ARCI-MBG measures, tramadol scores were lower than heroin but higher than MA, whereas the differences were not significant (*P* > 0.05). On the ARCI-PCAG measures, the scores produced by heroin were higher than those produced by tramadol (*P* < 0.05). However, the comparison with MA did not. On the ARCI-LSD measures, the scores of tramadol did not differ from other two active drugs (*P* > 0.05). 

## 4. Discussion

Using spontaneous data from the addiction unit we found that tramadol dependence fulfilling DSM-IV criteria occurs in 23 males with no known history of substance abuse. The findings indicated that tramadol appeared to produce high abuse liability within the long-term infrequent abuse by those without preexisting substance abuse and the high doses.

Tramadol is thought to have low potential for abuse, but the cases with no reported prior history of substance abuse became dependent on tramadol in our study. This may suggest that tramadol dependence in patients with no prior history of drug abuse differed from those of opiate addicts [21]. The results did not support the previous findings from experimental [22] and surveillance studies [23]. One of the possible reasons of conflicting results is the different study sample included in the study. As far as we know, this is the first study to evaluate the dependence and abuse liability of tramadol with no drug abuse history using multiple placebo as a preference. Thus we have no comparable studies to interpret and support our results. However, a wide abuse of tramadol does not largely appear to occur in other countries, and some controversy is driven by uncertainty in subpopulation vulnerable to tramadol dependence. Chinese tramadol abuse seems to be a growing problem and a unique phenomenon. We claimed that it may be due to a complex and polymorphic pharmacokinetic or pharmacodynamics characteristics or patient characteristics. The work of further exploring the effect of those factors in tramadol dependence is the guarantee for scientific and reasonable tramadol use. Another explanation may be a discrepancy between doses used in controlled studies and doses used in real life. The actual problem is that most tramadol abusers have a long duration of abusing drugs rather than short period in controlled study. Doses of tramadol in our sample extremely exceed the doses prescribed by the physician. Results from our study suggest that high dosage with a long abused time probably increase the ability of tramadol to induce dependence.

In this study, we examined the subjective effects of tramadol compared with multiple controls using ARCI-CV. There was good concordance between tramadol in general population in our study and the findings for this drug in opiate addicts [21] regarding its subjective effects. In particular, in the results of this study, particularly those for the comparison between tramadol and heroin, no statistically significant results were found on measures of MBG scale, which suggests that the euphoria effects of tramadol is somewhat qualitatively equal to heroin. As an example of the severity of tramadol dependence, this study implies that dependence of tramadol has led to admission to drug dependence clinic of hospital for detoxification or dose reduction. The proportion of subjects having at least one time or above detoxification treatment is 69.6% (16/23). Otherwise, neither animals experiment nor volunteer self-administration test has been associated with reports of actual abuse. Tramadol which did not produce a significant dependence effect and no abuse potential in the laboratory did not translate into actual abuse in the marketplace. Actual abuse of drugs is influenced by a number of factors that are not assessed in human laboratory studies of abuse potential, including the availability of other abused drugs, the cost or difficulty of obtaining the drug, expectations regarding social roles, and the potential consequences of abuse [24, 25]. In our study, it is possible that the doses much higher than the therapeutic range have contributed to its actual abuse. However, the association between higher dose and tramadol dependence should be further explored.

There were several factors that limit the interpretation regarding the significance of the results. First, we were not able to evaluate whether a dose response existed for the observed effects. Our results were limited to evaluating the higher doses of tramadol, which were higher than prescribed doses exceedingly. Second, as with any study of this type, conclusions drawn regarding the assessment of relative abuse potential for drugs depend on the patients' recollection. And the methodology is the major limitation of our study. We would improve and amend it in the following research. Third, the population tested in this study would not necessarily be expected to be representative of all tramadol abusers. Fourth, the sample is small. The reasons and its importance have been discussed above.

In conclusion, these results add further support to tramadol dependence data indicating that tramadol has a high risk of producing dependence potential. A history of tramadol abuse with a long period and/or high doses may be one of important risk factors for those with no prior drug abuse history. To further strengthen the surveillance and administration of tramadol would be necessary. 

## Figures and Tables

**Table 1:** Characteristics of demographic and history of drug use.

Variables	Tramadol (*n* = 23) *n* (%)	Heroin (*n* = 85) *n* (%)	MA^#^ (*n* = 60) *n* (%)	Healthy men (*n* = 50) *n* (%)	*F*/*χ* ^2^	*P*
Age (years)						
$\overset{-}{x}\pm s$	23.4 ± 4.1	25.7 ± 9.16	23.6 ± 8.1	22.9 ± 3.1	1.148	0.267
Range	(18–34)	(18–61)	(20–56)	(19–29)		
Education						
Junior high school	9 (39.1)	28 (32.9)	19 (31.7)	16 (32.0)	1.941	0.115
Senior high school and above	14 (60.9)	57 (67.1)	41 (68.3)	34 (68.0)		
Marital status						
Single	15 (65.2)	56 (65.9)	31 (51.7)	43 (86.0)	1.252	0.207
Others*	8 (34.8)	29 (34.1)	29 (48.3)	7 (14.0)		
Ethnicity						
Han ethnicity	21 (91.3)	70 (82.4)	59 (98.3)	49 (98.0)	1.094	0.236
Others	2 (8.7)	15 (17.6)	1 (1.7)	1 (2.0)		
Occupation						
Unemployed^*▼*^	7 (30.4)	31 (36.5)	19 (31.7)	30 (60.0)	0.289	0.591^#^
Employed	16 (69.6)	54 (63.5)	41 (68.3)	20 (40.0)		

^▼^Including students.

*Including married, divorced, and others.

^#^Methamphetamine.

**Table 2:** History of drug abuse of 23 tramadol abusers.

Variables	*N*
Duration of tramadol abuse (month)	
$\overset{-}{x}\pm s$	61.8 ± 30.2
Median (min–max)	63.0 (5–112)
Age of first tramadol use (year)	
$\overset{-}{x}\pm s$	18.3 ± 4.0
Median (min–max)	18.0 (13–28)
Purpose of first tramadol use	
Instigating by friends or other acquaintances	14
Curiosity or simulating	9
Medical purpose	2
Experienced the spiritual effects of drugs (euphoria)	2
Relieving negative emotions	2
Influence of family members	1
Route of administration	
Oral	23
Median dose of tramadol use (mg/per time)	
At the outset (range)	250.0 (50–1000)
Regular dose (range)	750.0 (100–5000)
Maximum (range)	2000.0 (250–10000)
Source of tramadol	
Private clinic or pharmacy	12
Normal pharmacy	6
Black market	6
Relatives or friends	1
Internet	1
Voluntary detoxification for one time or above	16

**Table 3:** Comparison of scores of ARCI scales across groups.

	Comparison	Mean value of difference	95% CI	*P*
Tramadol	MBG versus PCAG	2.43**	0.69, 4.18	0.007
	MBG versus LSD	2.30*	0.56, 4.05	0.010
	PCAG versus LSD	−0.13	−1.87, 1.61	0.882

MBG	Tramadol versus heroin	−0.27	−1.91, 1.37	0.745
	Tramadol versus MA	0.87	0.92, −0.97	0.348
	Tramadol versus healthy	2.58**	0.88, 4.28	0.004

PCAG	Tramadol versus heroin	−2.18**	−3.69, −0.68	0.005
	Tramadol versus MA	−0.19	−1.77, 1.38	0.807
	Tramadol versus healthy	2.02*	0.47, 3.57	0.012

LSD	Tramadol versus heroin	−0.58	−1.79, 0.62	0.334
	Tramadol versus MA	−2.14	−1.47, 1.04	0.733
	Tramadol versus healthy	1.50*	0.25, 2.75	0.020

LSD test, *P < 0.05, and **P < 0.01.
